# Morphological, physiochemical and antioxidant responses of *Maclura pomifera* to drought stress

**DOI:** 10.1038/s41598-019-55889-y

**Published:** 2019-12-17

**Authors:** Alireza Khaleghi, Rohangiz Naderi, Cecilia Brunetti, Bianca Elena Maserti, Seyed Alireza Salami, Mesbah Babalar

**Affiliations:** 1 Department of Horticultural Sciences, Faculty of Agriculture and Natural Resources, Arak University, Arak, Iran; 2 Department of Horticultural Sciences, College of Agriculture and Natural Resources, University of Tehran, Karaj, Iran; 3 National Research Council of Italy, Tree and Timber Institute (CNR-IVALSA), Sesto Fiorentino, Italy; 4 National Research Council of Italy, Institute of Sustainable Plant Protection (CNR-IPSP), Sesto Fiorentino, Italy

**Keywords:** Abiotic, Drought

## Abstract

Drought is one of the most important environmental factor limiting the growth of woody and non woody plants. In the present paper, we aimed to explore the performance of *Maclura pomifera* under a prolonged drought period followed by re-watering. *M. pomifera* plants were exposed to four different watering regimes (100%, 75%, 50% and 30% of the field capacity (FC)) for three weeks and then rewatered. The exposure to drought affected physiological, morphological and biochemical traits of *M. pomifera*. Leaf area, relative water content and water potential of leaf decreased in parallel with increased water deficit. Malondialdehyde content increased along with the drought stress experiment. Soluble carbohydrates (sucrose, glucose and fructose) accumulated during drought stress, but decreased after 22 days of water deficit in severe stressed plants (30% FC). Proline and mannitol, two compatible osmolytes, were higher in drought stresses plants than in control plants. Additionally the activity of antioxidant enzymes (SOD, APX, DHAR and GR) resulted affected by drought stress. In the recovery period, the physiological parameters as well as the proline content recovered at control levels, whereas soluble sugars, mannitol and total activity of antioxidant enzymes remained slight higher than in control plants, presumably to allow plants a complete recovery after stress. Our results suggest that *M. pomifera* has a good adaptive response to drought stress, probably corresponded to decreasing oxidative injury by induction of the antioxidant system and accumulation of stable and protective osmolytes such as proline and mannitol at higher rates.

## Introduction

Drought stress is one of the most important environmental challenge constraining plants living in arid- and semi-arid regions^1^. By the end of the 21^st^ century the drought-disaster risks are expected to increase because of the forecasted rising of temperature^2,3^. Researchers foretell that temperatures could increase by 3–9 °C by the end of the century with far-reaching effects. According to the Intergovernmental Panel on Climate Change 2014 (IPCC 2014) report, these negative effects are particularly exacerbated in mid-latitude and subtropical dry regions, where a significant reduction in mean precipitation and an increment in surface temperature are leading to the constant decline in agricultural land availability^4,5^. As a consequence, trees growth and viability in the forests and urban greenspace will be reduced^6,7^. Thus, the selection of plants tolerant to severe drought events and capable to recover afterwards is of crucial importance and the ability of individual tree species to cope with such environmental stresses needs to be considered in future silvicultural strategies^8^.

In plants, water shortage leads to the excessive production of reactive oxygen species (ROS) such as ^1^O_2_, O_2_^–^ and H_2_O_2_, which are very reactive and lead to rapidly injury to living tissues and macromolecules (e.g. DNA, lipids, proteins and carbohydrates), eventually resulting in induced programmed cell death (PCD) processes^9^. The risk of irrecoverable injuries within green tissues because of ROS production may increase under severe stress^7,10^. However, to compensate for their sessile lifestyle, plants have evolved many acclimation and adaptation mechanisms (i.e. antioxidant defense systems and osmotic adjustment) which may enhance their capability to survive and grow during short- and long-term drought stress^11,12^. The antioxidants enzymes, such as superoxide dismutase (SOD) and those belonging to Halliwell–Asada pathway, such glutathione reductase (GR), ascorbate peroxidase (APX), dehydroascorbate reductase (DHAR), and monodehydroascorbate reductase (MDHAR) play an important role in cleansing those activated oxygen species^9^. Regulation of the activity of these enzymes presumably is the main process in plant tolerance to environmental stresses^13,14^.

Sugars, (such as glucose, fructose and sucrose), sugar alcohols (such as mannitol) and amino acids (such as proline) accumulate under drought stress conditions in different plant species and function not only as osmolytes, but also as antioxidants, helping in ROS detoxification, membrane protection and enzyme/protein stabilization, ultimately improving plant resistance against abiotic stresses^15–17^.

*Maclura pomifera* (Raf.) Schneid. mainly known as Osage orange, belongs to Moraceae family and is native to the western Great Plains and Texas. *M. pomifera* is a long-lived, winter-deciduous and dioecious perennial hardwood tree species growing quickly to a height of 35 feet. *M. pomifera* has also many valuable characteristics: hardiness (wind-firm and resistance to breakage), ability to withstand repeated clipping, fast growing and resistance to diseases (termites and nematode)^18–20^. For these reasons, Osage orange is a tree that could be cultivated as a landscape plant and for afforestation. Recently, the fruit of *M. pomifera* has been investigated for biofuel production due to its high percentage of oil, fermentable sugars and other carbohydrates^21^. However, despite *M. pomifera* is considered a drought tolerant plants, by our knowledge there are no data available related to its response to different drought stress levels. Therefore, the aim of this work was to assess the response of *M. pomifera* plants to drought and their recovery after the stress determining the changes of water relations and stress biochemical markers, applying a randomized protocol based on the application of different levels of water shortage.

## Materials and Methods

### Plant material and treatments

The experiment was carried out on 4-year-old *Maclura pomifera* (Raf.) Schneid, saplings genotypes cultivated on a flat field in the Botanical Garden of University of Tehran, Karaj, Iran during summer 2013. Karaj city has a relatively dry and cold climate. The annual mean temperature is 14.4 °C, respectively. The maximum temperature may rise up to 42 °C in summer and may fall to −20 °C in winter. The average precipitation is around 247/3 mm and the annual relative humidity is 51%. During the experiment performance, the relative humidity and average precipitation were 38% and 00/0 mm. In early February 2013, seventy-two saplings were transplanted to 15-liter plastic pots (diameter 26 cm), containing a 2:1:1(V/V) mix of clay, sand and leaf composts. All saplings were irrigated every 2–3 days to soil field capacity until the onset of the experiments. All saplings were fertilized weekly with 20:20:20 N-P-K commercial fertilizer after the one month of growth starting. The experiment started on 5 September 2013.

*M. pomifera* saplings were arranged in four different watering regimes with a randomized block design as follows: one well-watered treatment [100% of field capacity (FC)] and three water-stressed treatments (75%, 50% and 30% of FC). First, all pots were watered to 100% FC and allowed to dehydrate by withholding water. It took 2, 5 and 7 days to reach the 75%, 50% and 30%, respectively. To attain the desirable moisture level in all treatment at the same time, dehydration time for each treatment was previously determined by pre-treatment before the onset of the experiment. Pre-treatment was conducted with 24 additional *M. pomifera* saplings, weighting the pots every day in order to estimate the dehydration time to reach the required field capacity. In the control treatment (100% FC), the pots were re-watered to 100% field capacity by replacing the amount of water transpired every day. In the water-stressed treatments, the pots were watered to 75%, 50% and 30% of FC every day to keep different drought levels in the soil. Soil water content was monitored every day by weighing pots. Drought stress was maintained for 22 days and then all saplings were watered at 100% field capacity. For each treatment, six saplings were used and leaf samples (five matured leaves per plant) from six different plants were collected at the 1, 8, 15 and 22th days from the beginning of the drought stress period and 1 and 7th days after re-watering. Leaf samples were frozen immediately in liquid nitrogen and then stored at −80 °C until used for further analyses.

### Determination of water relations parameters

#### Relative water content (RWC)

After leaf fresh weight determination, thirty matured leaves per each treatment were floated on deionised water for 24 h under low irradiance and low temperature (4 °C) and then turgid weights were calculated. Leaf dry weights were determined after oven-drying at 75 °C for 48 h. RWC was calculated according to Turner^22^, using the following formula:

$${\rm{RWC}}\,( \% )=[\frac{{\rm{Fresh}}\,{\rm{weight}}-{\rm{dry}}\,{\rm{weight}}}{{\rm{turgid}}\,{\rm{weight}}-{\rm{dry}}\,{\rm{weight}}}]\times 100$$

#### Leaf water potential and leaf area

Leaf water potential (Ψ_WP_; MPa) was measured by a pressure chamber (SKPM 1400; Skye Instruments, Llandrindod Wells, UK) at predawn. Leaf area (cm^2^) of six uppermost fully expanded leaf blades per sapling was determined by a Leaf Area Measurement System (Delta–T, England).

### Lipid peroxidation as malondialdehyde (MDA) equivalent

Lipid peroxidation was determined by estimating the TBA reactive substances (TBARS) as described by Hodges *et al*.^23^ with some modifications. In detail, 0.5 g of leaf sample were homogenized in 4 ml of 1% (w/v) trichloroacetic acid (TCA), and then centrifuged at 10,000 g for 10 min. Then, 1.5 ml of 0.5% (w/v) TBA in 20% (w/v) TCA was added to 1.5 ml of the supernatant. The mixtures were heated at 95 °C for 30 min and then quickly cooled in an ice bath. After centrifugation at 10,000 g for 5 min, the absorbance of the supernatant at 440, 532 and 600 nm was recorded. The concentration of TBARS (nmol g^−1^ DW) was calculated by using extinction coefficient of 155 mM^−1^ cm^−1^, and the results expressed as nmol MDA equivalents *per* grams.

### Measurement of proline content

Proline content was estimated following the method of Bates *et al*.^24^. Briefly, 20 mg of ground fresh leaves were mixed with 400 µl of ethanol and then heated at 85 °C in the block heater for 20 min. After cooling at room temperature, samples were centrifuged at 14,000 g for 5 min. The supernatant (50 µl) was mixed with 100 µl of reaction mixture (ninhydrin 1% (w/v) in acetic acid 60% (v/v), ethanol 20% (v/v)) and then heated at 95 °C in the block heater for 20 min. After cooling at room temperature, the mixtures were spin down quickly (1 min, 2500 rpm) and the solution absorbance was recorded at 520 nm. Proline concentration was calculated against a standard curve using 0.04 – 1 mM L-proline (Sigma, Milano, Italy).

### Sugar extraction and assay

Soluble carbohydrates were extracted and analyzed following the protocol of Tattini *et al*.^25^. Briefly, 5 ml of 75% ethanol was added to 200 mg of powdered fresh leaves. Samples were sonicated for 20 min. and centrifuged for 30 min at 10,000 g/min at room temperature to pellet the insoluble material. The supernatant was removed and the pellet was extracted twice as above. The ethanol fraction was reduced to dryness under vacuum and finally rinsed with 10 ml of water (pH 7.0). The aqueous extract was purified through –CH and –SAX Bond-Elute cartridges (Varian, Harbor City, CA, USA), and the eluate was reduced to dryness under vacuum at 35 °C. Samples were rinsed with 2 ml of ultrapure water and injected in a Series 200 HPLC equipped with 200-RI detector (Perkin Elmer), and separated on an 8 × 300 mm SC1011 column (Showa Denko, Tokyo, Japan) maintained at 88 ± 1 °C. Eluent was ultrapure water at a flow rate of 0.8 ml min^−1^. Sorbitol was added as internal standard.

### Measurement of antioxidant enzyme activities

#### Superoxide dismutase (SOD)

Fresh leaves (0.5 g) were homogenized in 500 µl of 0.15 M Tris-HCl buffer (pH 7.5), containing 50 mg polyvinylpyrrolidone (PVP) on ice and then centrifuged twice at 14000 *g* for 10 min at 4 °C. The supernatant was used for SOD activity assay.

Total SOD activity was determined by measuring the inhibition of photochemical reduction of nitro blue tetrazolium (NBT) as described by Giannopolitis and Rise^26^. One unit of SOD activity was defined as the amount of enzyme that inhibited 50% of NBT photoreduction monitored at 560 nm.

#### Ascorbate peroxidase (APX), dehydroascorbate reductase (DHAR) and glutathione reductase (GR) extraction

Fresh leaves (0.1 g) were homogenized in 200 µl of 50 mM K–phosphate buffer (pH 7.0) containing 50 mg of PVP, 0.1% Triton X-100, 10% glycerol, 5 mM ascorbic acid and 1 mM EDTA. After 30 min at 4 °C, the homogenate was centrifuged at 18000 *g* for 20 min at 4 °C. The supernatant was used for APX, DHAR and GR activity assay. To avoid enzyme inactivation all procedures for enzyme extraction and activity determination were carried out on ice bath.

#### APX determination

*APX* activity was measured following the method of Nakano and Asada^27^. To 100 µl of enzyme extract, 2.9 ml of reaction mixture containing 50 mM K–phosphate buffer (pH 7.0), 500 µM ascorbate (extinction coefficient, ε = 2.8 mM^−1^ cm^−1^), 100 µM EDTA and 100 µM H_2_O_2_ was added. The decrease in absorbance was recorded at 290 nm for 3 min. One enzyme unit was defined as µmol mg^−1^ protein oxidized ascorbate per min.

#### DHAR determination

DHAR activity was determined by measuring the reduction of dehydroascorbate (DHA) (ε = 14 Mm^−1^ cm^−1^) at 265 nm for 4 min, as described by Hossain and Asada^28^. 50 µL of enzyme extract was added to a reaction mixture containing 50 mM K–phosphate buffer (pH 7.0), 0.1 mM EDTA, 2.5 mM GSH and 2 mM DHA. One unit of DHAR activity was defined as the amount of enzyme that produces 1 nmol of AsA per min.

#### GR determination

GR activity was measured slightly modifying the method of Sofo *et al*.^29^, based on the rate of decrease in the absorbance of oxidized glutathione (GSSG), at 340 nm. The reaction mixture contained 0.1 M K–phosphate buffer (pH 7.0), 1.0 mM GSSG, 0.1 mM NADPH (dissolved in Tris–HCl buffer, pH 7.0) and 200 µl of enzyme extract in a total volume of 3.0 ml. An absorption coefficient of 6.22 mM^−1^cm^−1^ was used for calculations. One unit of GR activity was defined as the amount of enzyme that oxidizes 1 nmol of NADPH per min at 25 °C.

### Statistical analysis

All analyses were carried out on a randomized block design, in a factorial, with three replications (six saplings per replicate). Data were analyzed using repeated-measures ANOVA, with water treatment as between-subject effect and time as within subject effects. Significant differences among means were estimated at the 5% (P < 0.05) level, using Duncan test. All statistical analysis was performed using the SAS v. 9.1.3. software. In the figures, the spread of values is shown as error bars representing standard errors of the means.

## Results

### Effect of drought stress on growth rate

Growth of *M. pomifera* under drought stress was followed by measuring leaf area (LA), fresh weight (FW) and dry weight (DW) (Tables 1 and 2). The three parameters were not significantly affected by drought stress × time of treatment (Table 3). However, drought stress treatments significantly decreased FW, DW and LA. FW decreased by about 22%, 34% and 53% (p < 0.01), DW decreased about by 21%, 32% and 49% (p < 0.01), and LA was reduced about by 16% 18% and 34% (p < 0.01), at 75%, 50% and 30% of FC, respectively, as compared to the values measured in well-watered seedlings (control plants).

**Table 1:** The effect of drought stress treatments on fresh weight (FW), dry weight (DW) and leaf area (LA) of *M. pomifera* leaves. Means ± SE based on three replicates (*n* = 3) for FW, DW and LA are presented. Values followed by different letters are significantly different from each other at *p* < 0.05. FC, field capacity.

	Fresh weight (g)	Dry weight (g)	Leaf area (cm^2^)
100% FC (Control)	2.28489 ± 0.069a	0.8575 ± 0.026a	88.674 ± 2.02a
75% FC (mild stress)	1.77122 ± 0.064b	0.66906 ± 0.025b	74.57 ± 2.14b
50% FC (moderate stress)	1.49822 ± 0.075c	0.57683 ± 0.026c	72.563 ± 2.29b
30% FC (severe stress)	1.06178 ± 0.048d	0.4305 ± 0.014d	58.365 ± 1.57c

**Table 2:** The effect of drought stress treatments during experimental period on FW, DW and in leaves of *M. pomifera*. Data represents the average of three replicates. Vertical bars indicate ± SE. Values sharing a common letter are not significantly different at *p* < 0.01.

Drought treatment	Drought stress duration (day)	Fresh weight (g)	Dry weight (g)	Leaf area (cm^2^)
Control	**1-d**	2.10 ± 0.3 abcd	0.76 ± 0.09 bcde	82.7 ± 0.9 abcde
	**8-d**	2.01 ± 0.18 abcd	0.78 ± 0.06 bcd	82.6 ± 8.3 abcde
	**15-d**	2.32 ± 0.09 abc	0.87 ± 0.03 ab	94.5 ± 2.8 a
	**22-d**	2.50 ± 0.26 a	0.99 ± 0.08 a	93.8 ± 8.0 ab
	**23-d (rewatering)**	2.44 ± 0.49 ab	0.88 ± 0.17 ab	91.31 ± 2.3 abc
	**29-d (rewatering)**	2.32 ± 0.12 abc	0.84 ± 0.02 ab	86.88 ± 0.8 abcd
75% FC	**1-d**	1.77 ± 0.03 defg	0.66 ± 0.02 cdefgh	70.9 ± 0.4 efgh
	**8-d**	1.55 ± 0.08 efgh	0.58 ± 0.02 efghij	69.3 ± 1.7 efg
	**15-d**	1.80 ± 0.18 defg	0.67 ± 0.07 cdefg	82.3 ± 7.3 abcde
	**22-d**	1.83 ± 0.28 cdefg	0.69 ± 0.10 bcdef	77.8 ± 0.5 abcdef
	**23-d (rewatering)**	1.69 ± 0.29 defg	0.64 ± 0.11 defgh	69.7 ± 9.8 efgh
	**29-d (rewatering)**	1.97 ± 0.53 bcdef	0.75 ± 0.20 bcde	77.2 ± 5.0 bcdef
50% FC	**1-d**	1.63 ± 0.16 efg	0.61 ± 0.05 defghi	73.9 ± 4.6 def
	**8-d**	1.42 ± 0.2 ghij	0.59 ± 0.09 defghij	76.7 ± 7.9 cdef
	**15-d**	1.32 ± 0.21 ghijk	0.54 ± 0.08 fghijk	73.8 ± 5.98 def
	**22-d**	1.40 ± 0.27 ghijk	0.54 ± 0.09 fghijk	72.7 ± 7.4 defg
	**23-d (rewatering)**	1.47 ± 0.48 fgh	0.53 ± 0.18 fghijk	65.7 ± 7.8 efgh
	**29-d (rewatering)**	1.74 ± 0.51 defg	0.63 ± 0.18 defgh	72.3 ± 4.5 defg
30% FC	**1-d**	1.106 ± 0.1 hijk	0.47 ± 0.03 hijk	63.5 ± 6.4 fgh
	**8-d**	0.92 ± 0.1 jk	0.41 ± 0.07 ijk	61.0 ± 7.8 fgh
	**15-d**	0.88 ± 0.15 k	0.38 ± 0.07 k	56.5 ± 8.47 gh
	**22-d**	0.97 ± 0.12 ijk	0.41 ± 0.04 jk	56.0 ± 6.8 gh
	**23-d (rewatering)**	1.1 ± 0.11 hijk	0.41 ± 0.04 ijk	52.5 ± 5.6 h
	**29-d (rewatering)**	1.38 ± 0.11 ghijk	0.49 ± 0.03 ghijk	60.5 ± 2.5 fgh

**Table 3:** Repeated-measures ANOVA table of physiological and biochemical-related traits in *Maclura pommifera* leaves as affected by water supply (w) and sampling time (t).

Trait	F_w_	F_t_	F_w_ × _t_
FW	99.22**	3.80**	1.09 ns
DW	79.24**	1.80 ns	1.31 ns
LA	47.29**	1.19 ns	1.08 ns
RWC	144.25**	32.16**	8.53**
Ψ_W_	525.18**	129.21**	39.41**
MDA	6678.84**	117.74**	31.51**
proline	22.66**	7.96**	2.73**
Sucrose	81.43**	164.93**	53.03**
Glucose	37.29**	98.89**	43.87**
Fructose	2.20**	113.60**	34.22**
Mannitol	942.93**	142.75**	141.14**
SOD	2601.89**	116.09**	31.23**
APX	4557.91**	533.58**	185.41**
DHAR	2352.17**	84.98**	25.45**
GR	3357.42**	269.32**	65.61**

ns, not significant.

*P < 0.05.

**P < 0.01.

**Figure 1:**
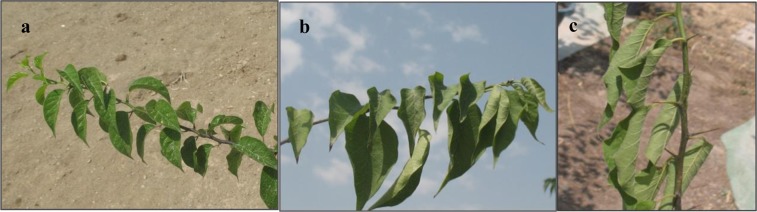
Leaf rolling of *M. pomifera* saplings under control (100% FC) (**a**), moderate (50% FC) (**b**) and severe (30% FC) (**c**).

**Figure 2:**
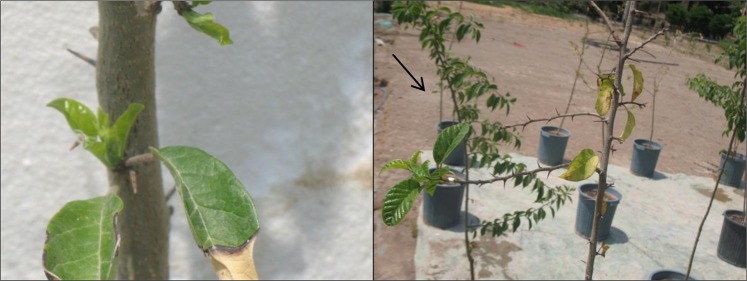
Left: Leaf abscission and yellowing observed under severe drought stress (30% FC); Right: new adaptive leaves re-sprouted by 18-d of drought stress in plants under severe drought stress (30% FC).

Leaf rolling of *M. pomifera* saplings was observed under moderate (50% FC) (left) and severe (30% FC) (right) drought stress (Fig. 1). Additionally, leaf abscission and yellowing were observed under severe drought stress, but new adaptive leaves re-sprouted by 18-d from the beginning of the experiment in plants under 30% FC (Fig. 2).

### Effect of drought stress on water relations parameters

**Figure 3:**
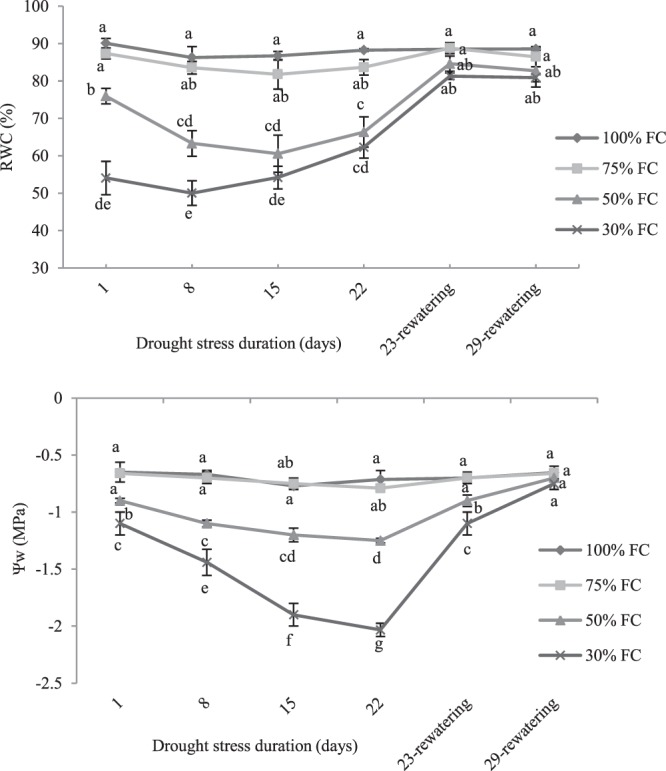
The effect of drought stress treatments on RWC and leaf water potential (Ψ_WP_) in leaves of *M. pomifera*. Data represents the average of three replicates. Vertical bars indicate ± SE. Values sharing a common letter are not significantly different at *p* < 0.01.

Relative leaf water content (RWC) of *M. pomifera* was significantly affected by water treatment. After 8-d of treatment, RWC declined by 26.58% and by 42.01% in plants exposed to 50% and 30% FC respectively, compared to control plants (100% FC) (Fig. 3). However, after 22-d of drought stress in plants experienced with 30% of FC, the RWC slightly increased respect to the values measured after 8-d from the beginning of stress. After re-watering, the leaf RWC was immediately restored indicating the rapid plant rehydration (Fig. 3).

The Ψ_WP_ was almost constant, ranging between −0.65 and −0.8 MPa, in control plants and in plants maintained at 75% of FC. The Ψ_WP_ at 50% FC and 30% FC decreased during drought stress progression (Fig. 3). Water potential decreased of about −1.32 MPa respect to control plants after 22-d of stress treatment in plants maintained at 30% FC. The Ψ_WP_ of drought-stressed plants completely restored to control values after of re-watering.

### Effect of drought stress on lipid peroxidation

**Figure 4:**
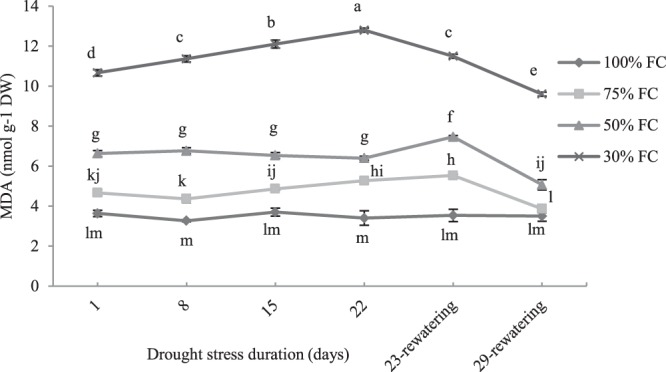
The effect of drought stress treatments on lipid peroxidation (MDA content) in leaves of *M. pomifera*. Data represents the average of three replicates. Vertical bars indicate ± SE. Values sharing a common letter are not significantly different at *p* < 0.01.

Malondialdehyde content, increased significantly under drought stress. The lowest MDA value was detected in the non-stressed plants and remained unchanged along with the experimental period. After 1-d of drought stress, MDA levels resulted increased of about 1.2-fold, 1.8-fold and 2.9-fold than control plants at 75%, 50% and 30% of FC, respectively. However, the concentration values remained quite constant along with the treatment in plants exposed to 75% and 50% FC, whereas in plants grown at 30% FC for 22-d, MDA reached the maximum value of 13 nmol g^−1^ DW. After re-watering, MDA content in plants exposed to 75% FC decreased to values similar to control plants, whereas significant differences respect to the levels measured in control conditions were maintained in plants subjected to 50% and 30% FC (Fig. 4).

### Effect of drought stress on proline content

**Figure 5:**
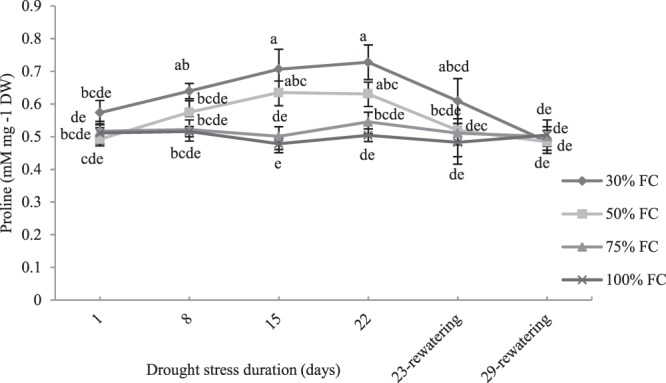
The effect of drought stress treatments on proline content in leaves of *M. pomifera*. Data represents the average of three replicates. Vertical bars indicate ± SE. Values sharing a common letter are not significantly different at *p* < 0.01.

The proline concentration slightly increased starting 8-d from the beginning of the experiment reaching values 1.2-fold and 1.4-fold higher than in control conditions in plants subjected to 50% and 30% FC respectively, at the end of the experiments. Proline concentration was not significantly affected by 75% FC. After re-watering, the proline content in plants exposed to drought resulted not significantly different from values detected in control plants (Fig. 5).

### Effect of drought stress on carbohydrates

The major carbohydrates detected in the leaves of *M. pomifera* were sucrose, fructose and glucose and the major sugar alcohol was mannitol.

**Figure 6:**
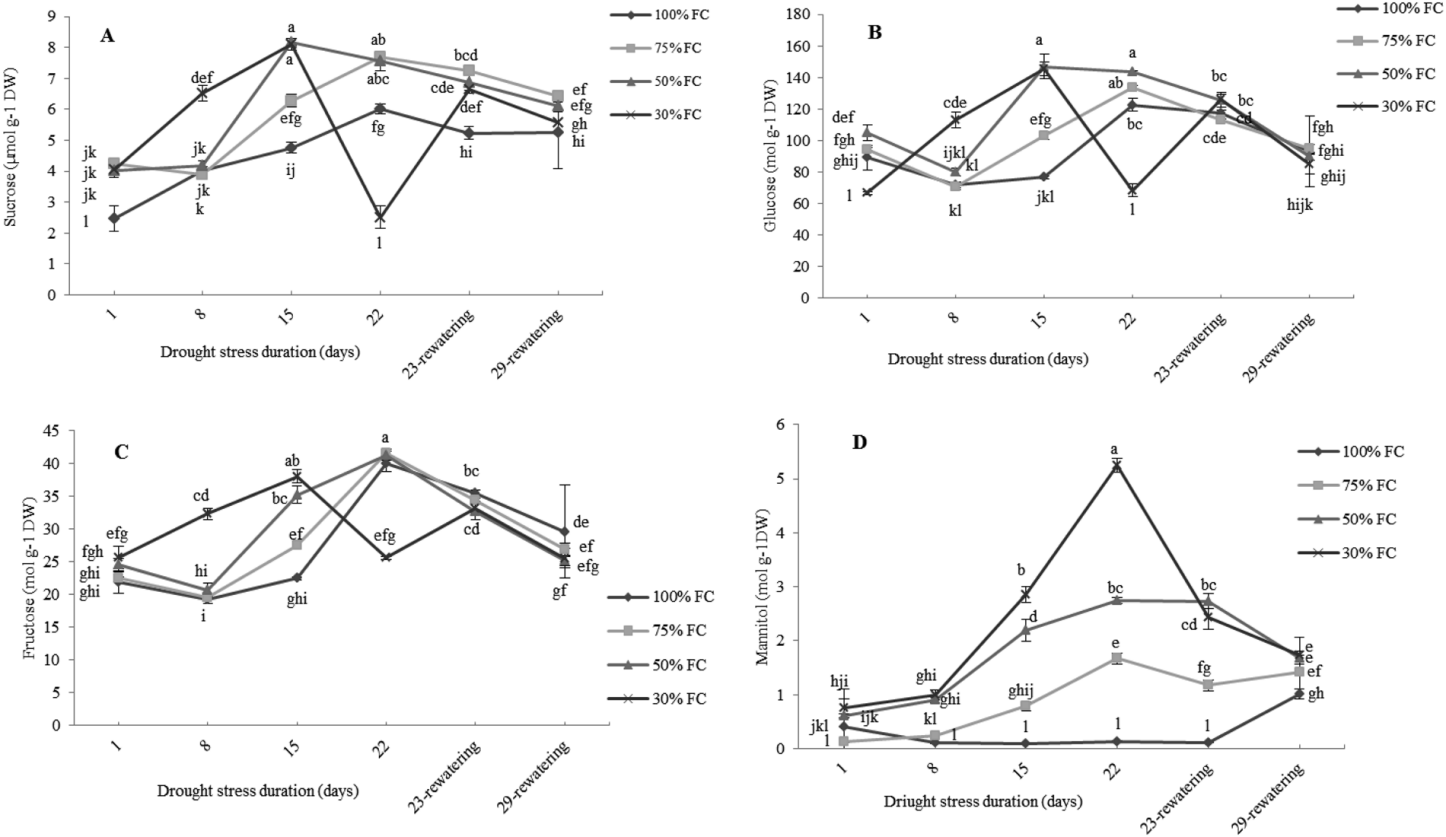
The effect of drought stress treatments on sucrose (**A**), glucose (**B**), fructose (**C**) and mannitol (**D**) concentrations in leaves of *M. pomifera*. Data represents the average of three replicates. Vertical bars indicate ± SE. Values sharing a common letter are not significantly different at *p* < 0.01.

The variation pattern of sucrose, glucose and fructose along with drought stress experiment was similar for all the three carbohydrates (Fig. 6A–C). The levels of sucrose, glucose and fructose significantly increased from the beginning of the experiment to 15-d in drought-stressed plants especially at 50% FC and 30% FC (Fig. 6A–C). Intriguingly after 22-d, plants in which water was reduced at 30% FC showed a consistent decline (p < 0.01) of sucrose (−140%), glucose (−44%) and fructose (−36%) content in comparison to those measured in the control plants. Conversely, the content of mannitol in leaves of drought-stressed plants of *M. pomifera* increased during the drought stress period and as a function of stress strength. In particular, at the end of the research (22-d), the mannitol values in drought-stressed plants were increased of about 13.4-fold, 22-fold and 42-fold at 75%, 50% and 30% FC, respectively, as compared to control plants (Fig. 6D).

One day after re-watering glucose and fructose of stressed-plants completely restored to values similar to control plants. Sucrose and mannitol content of drought-stressed plants restored 7-d after re-watering (29-d of time trial) (Fig. 6A–D).

### Effect of drought stress on enzyme activity

The activities of SOD, DHAR, APX and GR in the leaves of *M. pomifera* were significantly affected by the level of drought stress. In general, the activity of the enzymes linearly increased during the experiment, but the activity reached the maximum value in different times depending on the enzyme, and the activity of each enzyme did not arrived at the same levels of control plants after re-watering.

**Figure 7:**
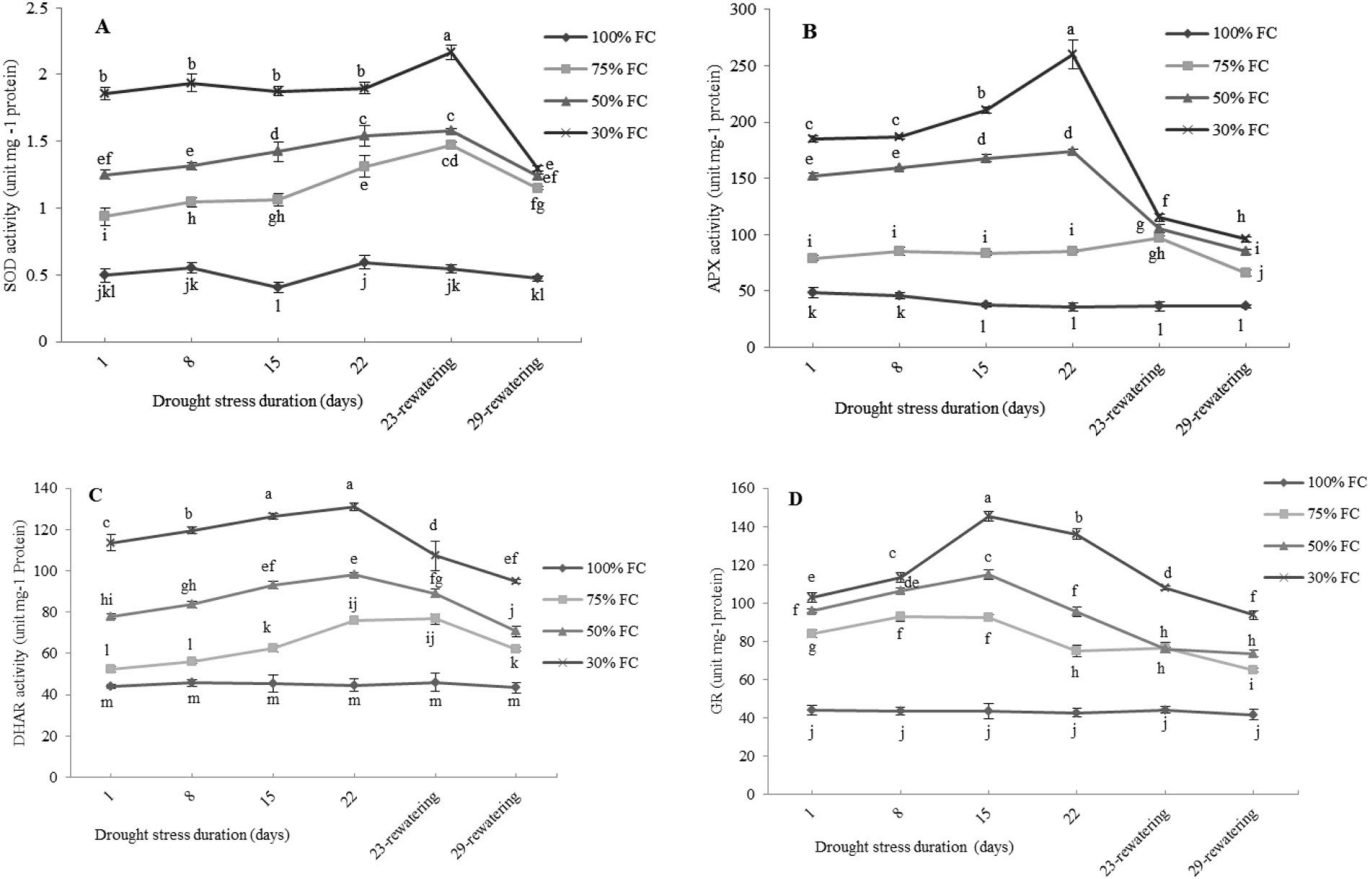
The effect of drought stress treatments on SOD (**A**), APX (**B**), DHAR (**C**) and GR (**D**) activities in leaves of *M. pomifera*. Data represents the average of three replicates. Vertical bars indicate ± SE. Values sharing a common letter are not significantly different at *p* < 0.01.

In particular, just after 1-d of drought stress, SOD activity increased 1.9-fold, 2.5-fold and 3.7-fold higher in plants at 75%, 50% and 30% of FC, respectively, compared to the values measured in control plants. The levels remained constant along with the drought experiment, but showed another significant increase one day after re-watering in 30% FC plants (Fig. 7A).

APX, DHAR and GR activities significantly and progressively increased after drought stress. The maximum level of 260 unit mg^−1^ protein for APX was observed after 22-d from the beginning of water withdraw (Fig. 7B), while DHAR and GR reached the maximum concentration value, 126 unit and 145 unit mg^−1^ protein, respectively after 15-d of drought stress (Fig. 7C,D).

## Discussion

### Osmotic effects of drought: role of proline and soluble carbohydrates

The decrease of growth, as FW, DW and LA, found in *M. pomifera* saplings was in agreement with the wastage in growth observed in *Jatropha curcas* and *Ziziphus rotundifolia*, two species growing in arid regions of the world^30,31^. In parallel to impairment of *M. pomifera* saplings growth, water stress strongly impacted leaf water status, inducing osmotic adjustments and leaf rolling. Leaf rolling is one of the morphological adaptations to drought because it decreases effective leaf area and transpiration^30^, and therefore it is a potentially drought avoidance mechanism in arid environments^32^. Additionally, *M. pomifera* saplings lost the major part of leaves in severe drought stress, but new adaptive leaves resprouted before the end of drought stress. Resprouting implies the remobilization and redistribution of belowground assimilates to support quick post disturbance regrowth and it is generally associated to a higher resistance of the plants to xylem cavitation^30,33–36^. On this basis, we hypothesized that in *M. pomifera* leaf shedding may not represent the negative reaction to extreme drought-stressed, but a morphological adaptation to reduce water loss, preserve xylem vessel from embolism and allow the redistribution of resources to protect bud bank^30,37^.

The decrease of water status in response to moderate (50% FC) and severe (30% FC) drought stress in *M. pomifera* has been previously reported also in other species such as *Populus kangdingensis*^38^, *Coffea canephora*^39^ and *Jatropha curcas* L.^40,41^. The slight increase of RWC in the leaves observed after 22-d, respect to the levels measured at 8-d from the beginning of the experiment, might be achieved by osmotic adjustment due to accumulation of proline, sucrose, glucose, fructose and mannitol^42^, which was observed in this work. Indeed, during the last fifteen days of drought treatment, the increase in RWC in plants exposed to 30% of FC was associated with a significant decrease in water potential, thus suggesting a role of osmotic compounds, mainly mannitol, in sustaining turgor dependent processes^43^.

The accumulation of MDA, a byproduct of oxidative damage to membrane lipids, in *M. pomifera* under water stress conditions is indicative of increased lipid peroxidation and it is in agreement with observation reported by several authors in different plant species^12,44–47^. The general increase in membrane lipids peroxidation is proportional to the intensity of drought stress and may derive from the spontaneous reactions of ROS with organic molecules contained in the membranes^9^. However, it cannot be excluded that a decrease in membrane fluidity^48^ and/or an increase in membrane leakiness may have concurred to increase lipid peroxidation^49^. However, after re-watering, the MDA content in leaves harvested from plants at 50% FC was only slightly higher than those measured in control conditions, thus suggesting a noticeable plasticity of *M. pomifera* metabolism to moderate water stress.

The increase of the proline concentration values in *M. pomifera* leaves in dependence on the time and drought level is in accordance with similar results reported for other plant species under water stress such as *Populus kangdingensis*^12^, *Arabidopsis thaliana*^50^ and *Capsicum annuum*^51^ and it may indicate an adaptive response to the imposed stress. Proline accumulation has been related to plant tolerance to drought stress, since proline is able to act as both an osmotic agent and a radical scavenger^9,12,15,52^. Proline transgenic wheat genotype was more tolerant towards water stress being more responsive to abscisic acid respect to the wild-type^53^. Proline, acting as an osmo-compatible solute and as a non-enzymatic antioxidant, can help decrease cell osmotic potential under stress conditions and stabilize proteins by preserving their chemical structure^52^. Proline may act also as a chelator of metal and a free radical scavenger, protecting leaves against lipid peroxidation^9^, which occurred in *M. pomifera* under drought stress as above discussed. In addition, proline has been recognized as a signaling molecule both by activating ROS detoxification pathways^54^ and by triggering specific gene expression to modulate mitochondrial functions under osmotic stresses^55^. The higher proline content found in plants exposed to severe and moderate stress condition may have played an essential role for plant recovery after stress.

The major leaf carbohydrates of *M. pomifera* were sucrose, fructose, glucose and mannitol. A relationship between drought stress tolerance and the accumulation of sugar alcohols (e.g. mannitol) and soluble carbohydrates has been previously reported^16^. The contents of the readily metabolizable carbohydrates, namely sucrose, glucose and fructose, significantly increased with drought stress progression, in accordance to similar observations in water-stressed *Quercus robur*^56^. In *M. pomifera* leaves, the found increase in glucose and fructose concentration under drought stress may derived from starch degradation. Similarly, it has been reported that an increment in hexoses concentration in pigeonpea leaves under drought was due to enhanced activities of enzymes hydrolyzing starch (e.g. amylase)^57^. These soluble carbohydrates not only provide osmotic adjustment, protect macromolecules (such as proteins) and membranes, but they can also fuel carbon for energetic metabolism when photosynthesis is reduced and play pivotal roles as signaling molecules, regulating biosynthesis and sensing of plant hormones^16,56,58–60^.

Interesting, the levels of mannitol significantly increased in leaves of *M. pomifera* during the drought stress treatments especially in severe drought stress (30% FC) and with increasing of drought duration, coinciding with the rapid decrease of mono and disaccharides levels. Indeed, the accumulation of mannitol under water stress conditions can diminish the negative effects of osmotic stresses^17,56,61^. The protective effect of mannitol is more stable than that of mono and disaccharides due to the formation of hydrogen bonds between osmolytes and macromolecules. This mechanism prevents the formation of intramolecular H-bonds, thus protecting the three-dimensional structures of macromolecules^42^. In addition, mannitol may play a possible function as a hydroxyl radical scavenger^62^. Supporting this hypothesis, mannitol is principally accumulated in plants growing in arid regions generally more adapted to drought and/or salt stress^63^.

### Regulation of antioxidant enzymes during drought stress progression

The exposure of plants to environmental stresses, including drought stress lead to over production of ROS^9,61^, such hydroxyl free radical (^•^OH), superoxide radical (O_2_^−^), singlet oxygen and hydrogen peroxide (H_2_O_2_) which are highly reactive and toxic molecules causing damage to nucleic acids, proteins, lipids, and carbohydrates^64^. To scavenge ROS, plants possess an efficient antioxidant defense system including non-enzymatic antioxidants and antioxidant enzymessuch as SOD, APX, DHAR, MDHAR, and GR^65,66^.

The first enzyme in the antioxidant pathway is SOD^9^ which removes superoxide radical by catalyzing its dismutation, one O_2_^•−^ being reduced to H_2_O_2_ and another oxidized to O_2_^67^. The increase of SOD activity observed in the leaves of *M. pomifera* as a function of the applied water stress levels might be correlated to enhanced protection from damages, among them lipid peroxidation, associated with oxidative stress, Transgenic rice plants overexpressing Mn-SOD1 showed less mitochondrial O_2_^•−^ under stress^68^. Significant increase in SOD activity under drought stress was also observed in *Picea asperata*^69^, *Citrus tangerine*^70^ and *Populus kangdingensis*^12^.

In *M. pomifera* under drought, the increased concentration of APX, DHAR and GR suggest the involvement of the Halliwell–Asada pathway, where APX reduces H_2_O_2_ to water and MDHA using ascorbic acid as substrate^61,71^. The stimulation of APX activity might be correlated to a possible increased H_2_O_2_ generation by the observed enhanced SOD activity. Yang *et al*.^69^ and Badawi *et al*.^72^ reported that APX activity increased in drought-stressed *Picea aspertata* and *Nicotiana tabacum*, respectively. Yang *et al*.^73^ observed increased APX activities in transgenic OsMT1 rice plants showing enhanced tolerance to drought.

Ascorbate (AsA) is a potent antioxidant molecule which protects plants against oxidative damage imposed by environmental stresses, such as drought and ozone^74,75^. DHAR is responsible for regenerating AsA from the oxidized state and regulates the cellular AsA redox state, which is crucial in the response to abiotic stresses^9,76^. Transgenic tobacco plants overexpressing cytosolic DHAR gene from *Arabidopsis thaliana* showed maintenance of AsA redox status and exhibited tolerance to salt, drought, polyethylene glycol and ozone stresses^74^. The higher AsA level and APX activity in DHAR-overexpressing transgenic tobacco contributed to their increased capacity of antioxidant and tolerance to environmental stresses^77^. Arabidopsis mutant with a deficient cytosolic DHAR (*AtDHAR3* mutant), which completely lacked cytosolic DHAR activity, was highly sensitive to environmental ozone stress, suggesting that ASH recycling is important in responding to environmental ROS^78^.

GR plays a key role in defense system by sustaining the reduced status of glutathione (GSH) a disulphide reductant which protects thiol groups of enzymes, regenerates ascorbate and reacts with singlet oxygen and hydroxyl radicals^13,79^. GSH and GR play a key role in determining the tolerance of a plant under various stresses^9^. This might be due to maintain a high ratio of NADP^+^/NADPH, therefore ensuring availability of NADP + for accepting electrons from photosynthetic electron transport chain and facilitating the regeneration of oxidized ascorbate^44^.The activities of GR under drought stress conditions were enhanced in various plants, e.g., diploid hybrid *Pinus densata*^80^, perennial xerophyte *Capparis ovata*^44^ and *Ctenanthe setosa*^32^ and *Picea asperata* seedlings^69^. Overall the observed noticeable increased efficiency of the antioxidant enzyme complex in *M. pomifera* under drought stress might be correlated to tolerance mechanisms based on fine-tuned regulation of the its redox status.

### Impact of re-watering on *M. pomifera* under drought stress

The recovery of the physiological parameters such as RWC and leaf water potential, as well as, the sprouting of new leaves, claims for a sort of capability of *M. pomifera* to withstand drought stress. However, as the lipid peroxidation indicated by MDA content and the activities of several antioxidant enzymes, namely SOD, DHAR and GR remained higher in drought stressed plants (30% FSW) than in control ones suggest that the recovery extent might be correlated to the intensity of the stress^81^.

## Conclusion

This paper presented a study focused to measure several biochemical and physiological parameters in the leaves of *M. pomifera* experienced with different levels of drought stress and re-watering in the natural environment. Overall, saplings of *M. pomifera* displayed a certain morphological plasticity suggesting a possible tolerance to drought stress by re-balancing soil water uptake and canopy water loss, by reducing LA, Ψ_WP_ and increasing of osmotic compounds such as proline and mannitol. Our data suggest that drought tolerance of *M. pomifera* might be correlated with diminishing oxidative damage by activation of the antioxidant systems. Finally, this species might be resistant to subsequent drought cycles because of its capacity to recover after re-watering. Further experiments should be undertaken in consecutive cycles of drought and re-watering (recovery) to definitely confirm the possible use of *M. pomifera* as suitable plants for urban greenspace.

## References

[1] Chaves MM, Maroco JP, Pereira JS (2003). Understanding plant responses to drought-from genes to the whole plant. Funct. Plant Biol..

[2] IPCC. Executive summary of the Intergovernmental Panel on Climate Change, February 2007, www.ipcc.com.ch (2007).

[3] Li YP, Ye W, Wang M, Yan X (2009). Climate change and drought: a risk assessment of crop-yield impacts. Clim. Res..

[4] Wang W, Vinocur B, Altman A (2003). Plant responses to drought, salinity and extreme temperatures: towards genetic engineering for stress tolerance. Planta..

[5] Barros, V. R. *et al*. *Climate change 2014: impacts, adaptation, and vulnerability. Part B: regional aspects. Contribution of Working Group II to the fifth assessment report of the Intergovernmental Panel on Climate Change*. (Cambridge University Press, 2014).

[6] Ciais P (2005). Europe-wide reduction in primary productivity caused by the heat and drought in 2003. Nature..

[7] Galle A, Feller U (2007). Changes of photosynthetic traits in beech saplings (*Fagus sylvatica*) under severe drought stress and during recovery. Physiol. Plant..

[8] Arend M, Kuster T, Günthardt-Goerg MS, Dobbertin M (2011). Provenance-specific growth responses to drought and air warming in three European oak species (*Quercus robur, Q. petraea and Q. pubescens*). Tree Physiol..

[9] Gill SS, Tuteja N (2010). Reactive oxygen species and antioxidant machinery in abiotic stress tolerance in crop plants. Plant Physiol. Bioch..

[10] Reddy AR, Chaitanya KV, Vivekanandan M (2004). Drought-induced responses of photosynthesis and antioxidant metabolism in higher plants. J. Plant Physiol..

[11] Fu J, Huang B (2001). Involvement of antioxidants and lipid peroxidation in the adaptation of two cool-season grasses to localized drought stress. Environ. Exp. Bot..

[12] Yin C, Peng Y, Zang R, Zhua Y, Li C (2005). Adaptive responses of *Populus kangdingensis* to drought stress. Physiol. Plant..

[13] Noctor G, Foyer CH (1998). Ascorbate and glutathione: keeping active oxygen under control. Annu. Rev. Plant Physiol. Plant Mol. Biol..

[14] Sairam RK, Chandrasekhar V, Srivastava GC (2001). Comparison of hexaploid and tetraploid wheat cultivars in their responses to water stress. Biol. Plantarum..

[15] Ashraf M, Foolad MR (2007). Roles of glycine betaine and proline in improving plant abiotic stress resistance. Environ. Exp. Bot..

[16] Bartels D, Sunkar R (2005). Drought and salt tolerance in plants. Crit. Rev. Plant Sci..

[17] Seki M, Umezawa T, Urano K, Shinozaki K (2007). Regulatory metabolic networks in drought stress responses. Plant Biol..

[18] Smith JL, Perino JV (1981). Osage Orange (*Maclura pomifera*): History and economic uses. Econ. Bot..

[19] Santamour FS, Riedel LGH (1993). Susceptibility of various landscape trees to root-knot nematodes. J. Arboric..

[20] Rudenskaya GN, Bogdanova EA, Revina LP, Golovkin BN, Stepanov VM (1995). Macluralisin- a serine proteinase from fruits of *Maclura pomifera* (Raf.) Schneid. Planta.

[21] Saloua F, Saber C, Hedi Z (2010). Methyl ester of [*Maclura pomifera* (Rafin.) Schneider] seed oil: Biodiesel production and characterization. Bioresour. Technol..

[22] Turner NC (1981). Techniques and experimental approaches for the measurement of plant water status. Plant Soil..

[23] Hodges DH, DeLong JM, Forney CF, Prange RK (1999). Improving the thiobarbituric acid-reactive-substances assay for estimating lipid peroxidation in plant tissues containing anthocyanin and other interfering compounds. Planta.

[24] Bates LE, Waldren RP, Teare ID (1973). Rapid determination of free proline for water stress studies. Plant Soil..

[25] Tattini M, Gucci R, Romani A, Baldi A, Everard JD (1996). Changes in non-structural carbohydrates in olive (*Olea europaea*) leaves during root zone salinity stress. Physiol. Plant..

[26] Giannopolitis CN, Reis SK (1977). Superoxide dismutase I. Occurrence in higher plants. Plant Physiol..

[27] Nakano Y, Asada K (1981). Hydrogen peroxide is scavenged by ascorbate-specific peroxidase in spinach chloroplasts. Plant Cell Physiol..

[28] Hossain MA, Asada K (1984). Purification of dehydroascorbate reductase from spinach and its characterization as a thiol enzyme. Plant Cell Physiol..

[29] Sofo A, Tuzio AC, Dichio B, Xiloyannis C (2005). Influence of water deficit and rewatering on the components of the ascorbate–glutathione cycle in four interspecific *Prunus* hybrids. Plant Sci..

[30] Arndt SK, Clifford SC, Wanek W, Joness HG, Popp M (2001). Physiological and morphological adaptations of the fruit tree *Ziziphus rotundifolia* in response to progressive drought stress. Tree Physiol..

[31] Silva EN, Ribeiro RV, Ferreira-Silva SL, Viegas RA, Silveira JAG (2010). Comparative effects of salinity and water stress on photosynthesis, water relations and growth of *Jatropha curcas* plants. J. Arid Environ..

[32] Saruhan N, Terzi R, Saglam A, Kadioglu A (2009). The relationship between leaf rolling and Ascorbate-Glutathione cycle enzymes in apoplastic and symplastic areas of *Ctenanthe setosa* subjected to drought stress. Biol. Res..

[33] Moreira B, Tormo J, Pausas JG (2012). To resprout or not to resprout: factors driving intraspecific variability in resprouting. Oikos..

[34] Schwilk DW, Ackerly DD (2005). Is there a cost to resprouting? Seedling growth rate and drought tolerance in sprouting and nonsprouting *Ceanothus* (Rhamnaceae). Am. J. Bot..

[35] Klimes JO, Klimes L (2007). Bud banks and their role in vegetative regeneration–a literature review and proposal for simple classification and assessment. Perspect. Plant Ecol. Evol. Syst..

[36] Pausas JG (2016). Towards understanding resprouting at the global scale. New Phytol..

[37] Munné-Bosch S, Alegre L (2004). Die and let live: leaf senescence contributes to plant survival under drought stress. Funct. Plant Biol..

[38] Yang F, Miao LF (2010). Adaptive responses to progressive drought stress in two poplar species originating from different altitudes. Silva Fenn..

[39] Ana Lucia S (2002). Photochemical responses and oxidative stress in two clones of *Coffea canephora* under water deficit conditions. Environ. Exp. Bot..

[40] Fini A, Bellasio C, Pollastri S, Tattini M, Ferrini F (2013). Water relations, growth, and leaf gas exchange as affected by water stress in *Jatropha curcas*. J. Arid Environ..

[41] Maes WH (2009). Plant–water relationships and growth strategies of *Jatropha curcas* L. seedlings under different levels of drought stress. J. Arid Environ..

[42] Chaves MM, Oliveira MM (2004). Mechanisms underlying plant resilience to water deficits: prospects for water-saving agriculture. J. Exp. Bot..

[43] Fini A (2012). Drought stress has contrasting effects on antioxidant enzymes activity and phenylpropanoid biosynthesis in Fraxinus ornus leaves: An excess light stress affair?. J Plant Physiol..

[44] Ozkur O, Ozdemir F, Bor M, Turkan I (2009). Physiochemical and antioxidant responses of the perennial xerophyte Capparis ovata Desf. to drought. Environ. Exp. Bot..

[45] Gaber MA (2011). Differential regulation of photorespiratory gene expression by moderate and severe salt and drought stress in relation to oxidative stress. Plant Sci..

[46] Catola S (2016). Physiological and metabolomic analysis of Punica granatum (L.) under drought stress. Planta..

[47] Santana Souza Vieira DD (2016). Polyploidization alters constitutive content of volatile organic compounds (VOC) and improves membrane stability under water deficit in Volkamer lemon (Citrus limonia Osb.) leaves. Environ. Exp. Bot..

[48] Xu L, Han L, Huang B (2011). Membrane fatty acid composition and saturation levels associated with leaf dehydration tolerance and post-drought rehydration in Kentucky bluegrass. Crop sci..

[49] Beck EH, Fettig S, Knake C, Hartig K, Bhattarai T (2007). Specific and unspecific responses of plants to cold and drought stress. J. Biosci..

[50] Sperdouli I, Moustakas M (2012). Interaction of proline, sugars, and anthocyanins during photosynthetic acclimation of Arabidopsis thaliana to drought stress. Plant Physiol..

[51] Anjum SA, Farooq M, Xie X, Liu X, Ijaz MF (2012). Antioxidant defense system and proline accumulation enables hot pepper to perform better under drought. Sci. Hortic..

[52] Kauer G, Asthir B (2015). Proline: a key player in plant abiotic stress tolerance. Biol. Plantarum..

[53] Nayyar H, Walia DP (2003). Water stress induced proline accumulation in contrasting wheat genotypes as affected by calcium and abscisic acid. Biol. Plantarum..

[54] Hossain, M. A., Hoque, M. A., Burritt, D. J., & Fujita, M. Proline protects plants against abiotic oxidative stress: biochemical and molecular mechanisms. In Oxidative damage to plants. 477–522 (Academic Press: Cambridge, MA, USA, 2014).

[55] Anjum SA (2011). Morphological, physiological and biochemical responses of plants to drought stress. Afr. J. Agric. Res..

[56] Spieß N (2012). Ecophysiological and transcriptomic responses of oak (Quercus robur) to long-term drought exposure and rewatering. Environ. Exp. Bot..

[57] Keller F, Ludlow MM (1993). Carbohydrate metabolism in drought-stressed leaves of pigeonpea (Cajanus cajan). J. Exp. Bot..

[58] Koch K (2004). Sucrose metabolism: regulatory mechanisms and pivotal roles in sugar sensing and plant development. Curr. Opin. in Plant Biol..

[59] Rolland F, Baena-Gonzalez E, Sheen J (2006). Sugar sensing and signaling in plants: conserved and novel mechanisms. Annu. Rev. Plant Biol..

[60] Tattini M (2014). Isoprene production in transgenic tobacco alters isoprenoid, non-structural carbohydrate and phenylpropanoid metabolism, and protects photosynthesis from drought stress. Plant Cell Environ..

[61] Koyro, H., Ahmad, P. & Geissler, N. Abiotic stress responses in plants: An overview. Environmental adaptations and stress tolerance of plants in the era of climate change, 1–28 (Springer, 2012).

[62] Shen B, Jensen RG, Bohnert HJ (1997). Mannitol protects against oxidation by hydroxyl radicals. Plant physiol..

[63] Merchant A, Tausz M, Arndt SK, Adams MA (2006). Cyclitols and carbohydrates in leaves and roots of 13 Eucalyptus species suggest contrasting physiological responses to water deficit. Plant Cell Environ..

[64] Mittler R, Vanderauwera S, Gollery M, Van Breusegem F (2004). Reactive oxygen gene network of plants. Trends plant sci..

[65] Blokhina O, Virolainen E, Fagerstedt KV (2003). Antioxidants, oxidative damage and oxygen deprivation stress: a review. Ann. Bot..

[66] Palma JM (2006). Antioxidative enzymes from chloroplasts, mitochondria, and peroxisomes during leaf senescence of nodulated pea plants. J. Exp. Bot..

[67] Halliwell B (2006). Reactive species and antioxidants. redox biology is a fundamental theme of aerobic life. Plant Physiol..

[68] Li CR (2013). Unravelling mitochondrial retrograde regulation in the abiotic stress induction of rice ALTERNATIVE OXIDASE 1 genes. Plant Cell Environ..

[69] Yang Y, Han C, Liu Q, Lin B, Wang J (2008). Effect of drought and low light on growth and enzymatic antioxidant system of Picea asperata seedlings. Acta physiol. Plant..

[70] Wu QS, Zou YN, Xia RX (2006). Effects of water stress and arbuscular mycorrhizal fungi on reactive oxygen metabolism and antioxidant production by citrus (Citrus tangerine) roots. Eur. J. Soil Biol..

[71] Mittler, R. & Poulos, T. L. Ascorbate peroxidase. In: Smirnoff N (ed) Antioxidants and reactive oxygen species in plants. 87–100 (Blackwell Publishing, Oxford, 2005).

[72] Badawi GH (2004). Enhanced tolerance to salt stress and water deficit by overexpressing superoxide dismutase in tobacco (Nicotiana tabacum) chloroplast. Plant Sci..

[73] Yang Z, Wu Y, Li Y, Ling HQ, Chu C (2009). OsMT1a, a type 1 metallothionein, plays the pivotal role in zinc homeostasis and drought tolerance in rice. Plant Mol. Biol..

[74] Eltayeb AE (2006). Enhanced tolerance to ozone and drought stresses in transgenic tobacco overexpressing dehydroascorbate reductase in cytosol. Physiol. Plant..

[75] Foyer CH, Noctor G (2011). Ascorbate and glutathione: the heart of the redox hub. Plant Physiol..

[76] Chen Z, Gallie DR (2006). Dehydroascorbate reductase affects leaf growth, development, and function. Plant Physiol.

[77] Yin L (2010). Overexpression of dehydroascorbate reductase, but not monodehydroascorbate reductase, confers tolerance to aluminum stress in transgenic tobacco. Planta..

[78] Yoshida S (2006). Cytosolic dehydroascorbate reductase is important for ozone tolerance in Arabidopsis thaliana. Plant Cell Physiol..

[79] Ahmad P, Jaleel CA, Salem MA, Nabi G, Sharma S (2010). Roles of enzymatic and non-enzymatic antioxidants in plants during abiotic stress. Crit. Rev. Biotechnol..

[80] Gao D (2009). Physiological responses to gradual drought stress in the diploid hybrid Pinus densata and its two parental species. Trees..

[81] Xu Z, Zhou G, Shimizu H (2010). Plant responses to drought and rewatering. Plant Signal Behav. Jun.

